# Positive Childhood Experiences, Cognition, and Biomarkers of Alzheimer’s Disease

**DOI:** 10.3390/ijerph22040525

**Published:** 2025-03-30

**Authors:** Joshua H. Owens, Charles C. Windon, Dan Mungas, Rachel A. Whitmer, Paola Gilsanz, Jennifer J. Manly, M. Maria Glymour

**Affiliations:** 1 Department of Clinical and Health Psychology, University of Florida, 1225 Center Dr, Gainesville, FL 32603, USA; jowens1@phhp.ufl.edu; 2 Memory and Aging Center, Department of Neurology, Weill Institute for Neurosciences, University of California, 675 Nelson Rising Lane, Suite 190, San Francisco, CA 94158, USA; 3 Department of Neurology, University of California Davis, 4860 Y St., Suite 3900, Sacramento, CA 95817, USA; dmmungas@ucdavis.edu; 4 Division of Epidemiology, Public Health Sciences, School of Medicine, University of California Davis, One Shields Avenue, Davis, CA 95616, USA; rawhitmer@ucdavis.edu; 5 Division of Research, Kaiser Permanente Oakland, 2000 Broadway, Oakland, CA 94612, USA; paola.gilsanz@kp.org; 6 Taub Institute for Research on Alzheimer’s Disease and the Aging Brain, Columbia University, 630 West 168th Street, P&S Box 16, New York, NY 10032, USA; jjm71@cumc.columbia.edu; 7 Department of Epidemiology, Boston University School of Public Health, 715 Albany Street, Boston, MA 02118, USA; mglymour@bu.edu

**Keywords:** childhood experiences, social determinants, cognition, amyloid, MRI biomarkers, diverse populations

## Abstract

Positive childhood experiences (PCEs) have unknown effects on late life cognition and Alzheimer’s Disease biomarkers. We examined 406 Asian, 1179 Black, 349 Latinx, and 498 White KHANDLE and STAR study participants with data on PCEs, longitudinal cognitive measures, MRI (n = 560), and amyloid PET (n = 281). We conducted mediation and multigroup models within the structural equation modeling framework allowing us to examine the direct association of PCEs with episodic memory level and change as well as the indirect effects of PCEs through education. We additionally conducted linear regressions examining the association of PCEs with MRI and amyloid PET outcomes. Average participant age was 74 (53–90) and 62% were female. Overall, PCEs were positively associated with memory intercept and change. Education significantly mediated the association between PCEs and memory intercept. PCEs were not associated with hippocampal volume or amyloid burden in the combined sample or across individual ethnocultural groups. PCEs are positively related to episodic memory through the promotion of educational attainment.

## 1. Introduction

Inequities in dementia incidence and prevalence are well-documented and may be partly attributable to early-life experiences. Exposure to adverse childhood experiences (ACEs) has a deleterious impact on late-life cognition [1,2,3,4], though certain ACEs may confer greater risk of Alzheimer’s Disease and Related Dementias (AD/ADRD) than others [5]. In contrast, exposure to positive factors like education [6] and higher socioeconomic status (SES) environments [7] in childhood may benefit late-life cognition. A growing body of literature has begun to examine these positive, health promoting factors.

Positive childhood experiences (PCEs) are not well defined in the literature compared to ACEs. One proposed definition of PCEs is “essential, interrelated experiences that engage the child, the parent, and the parent-child relationship in order to achieve the designated child health outcomes” [8]. PCEs include, but are not limited to safe and stable environments, nurturing relationships, and opportunities for constructive social engagement [8,9,10]. In contrast, ACEs have previously been defined as “childhood events, varying in severity and often chronic, occurring within a child’s family or social environment that cause harm or distress, thereby disrupting the child’s physical and psychological health and development” [11]. Both ACEs and PCEs have associations with health outcomes in adulthood. Lee et al. [12] demonstrated that exposure to high levels of childhood family-life happiness predicted cognitive functioning in older adulthood in one of the few studies that examined the relationship between PCEs and cognition [13,14]. Other literature has shown that PCEs are also associated with better cardiovascular and mental health in adulthood [15,16] and can partially buffer the negative health effects of ACEs [13]. More research is needed to determine if these benefits on health in mid-life also coincide with improved cognition in older adulthood. It also remains unclear whether PCEs provide protection equally across different ethnocultural groups, given the impact of systemic racism and racial biases that disproportionately impact historically excluded populations.

In the United States, historical injustices have led to inequities in risk factors for AD/ADRD between ethnocultural groups. Individuals racialized as Black and Latino/a/x (heretofore Latinx) disproportionately face barriers in accessing quality healthcare services, have higher rates of chronic disease, and experience poorer health outcomes. These disparities are driven in large part by Social Determinants of Health (SDH). Black and Latinx individuals also have less access to quality education and are more likely to live in more disadvantaged neighborhoods than their White counterparts [17,18,19]. Black, Latinx, and Asian American individuals more commonly encounter race-based interpersonal discrimination in their day-to-day environment and when seeking healthcare [12]. PCEs may serve as a resilience factor that partially buffers the deleterious effects of SDH and systemic factors, but it is possible that the relationship between PCEs and cognition or brain health in older adulthood may differ among ethnocultural groups given the diverse lived experiences of these individuals.

Mechanistically, the impact of PCEs on AD/ADRD risk is incompletely clear. PCEs likely positively impact adult health behaviors [20,21,22], resulting in lower risk of AD/ADRD in late age. One key pathway that may link PCEs to late-life cognitive outcomes is education. PCEs often encompass supportive school environments and access to educational support at home; thus, it is reasonable to infer that they play a role in shaping an individual’s educational attainment. In support of this, Crouch et al. [23] found a positive association between PCEs and educational success, suggesting that education may serve as a partial mediator between PCEs and cognitive outcomes in adulthood. Furthermore, higher educational quality and greater years of schooling are well-established contributors to cognitive resilience in older adulthood. Concurrently, decreased exposure to ACEs may reduce brain vulnerability in late age. Prior work has demonstrated hippocampal development in children is impacted by childhood SES [24] and hippocampal volume is lower among adults who have experienced childhood mistreatment [25,26]. Given the hippocampus’s critical role in memory and its susceptibility to neurodegenerative processes, early-life experiences—both positive and negative—may have lasting implications for brain health. Relationships between childhood experiences, positive and negative, and other MRI measures of Alzheimer’s Disease as well as amyloid pathology are largely unknown.

Questions remain regarding the impact of PCEs on late-life cognition and the underlying mechanism for reduced AD/ADRD risk, particularly among ethnoculturally diverse populations. The current study had several aims: (1) examine the association of PCEs with episodic memory, and cognitive change in a diverse cohort of older adults, (2) examine education as a potential mediator between PCEs and episodic memory, (3) determine if this relationship differs between ethnocultural groups, (4) and examine the association between PCEs and imaging (hippocampal volume and amyloid burden) biomarkers of AD/ADRD. We examined this topic within a longitudinal study of diverse older adults and hypothesized that more self-reported PCEs would be associated with better cognitive performance in verbal episodic memory, and that this relationship would be mediated by education. Further, it was hypothesized that more self-reported PCEs would be associated with less neurodegeneration (as measured by hippocampal volume), and less amyloid burden (as defined by amyloid PET), but that these associations would differ between racial and ethnic groups.

## 2. Materials and Methods

### 2.1. Study Population

Data used for this project were derived from the Kaiser Study of Healthy Aging and Diverse Life Experiences (KHANDLE) and Study of Healthy Aging in African Americans (STAR) cohorts. KHANDLE is a longitudinal aging study of ethnoculturally diverse community-dwelling adults aged 65 or older (as of 1 January 2017) who are residents within the San Francisco Bay Area and Sacramento Area in Northern California. KHANDLE eligible participants must be members of the Kaiser Permanente Northern California health care system, a large, integrated health maintenance organization, and be English or Spanish speaking [27]. Individuals volunteer to participate in the KHANDLE study. The STAR cohort is comprised of community-dwelling individuals who self-identify as African American or Black and are members of Kaiser Permanente Northern California. STAR eligible individuals must be aged 50 or older (as of 1 January 2018) [28]. Individuals volunteer to participate in the STAR study. Exclusionary criteria for KHANDLE and STAR include: (1) electronic medical record diagnosis of dementia or neurodegenerative condition (Parkinson’s disease with dementia, Huntington’s disease, Lewy Body disease, Frontotemporal dementia); (2) presence of health conditions interfering with participation in study interviews (i.e., hospice activity in previous 12 months, severe chronic obstructive pulmonary disease in previous 6 months, congestive heart failure hospitalizations within previous 6 months, end-stage renal disease or dialysis within previous 12 months).

For the current project, the sample initially consisted of 2471 individuals from KHANDLE and STAR. Three individuals were excluded due to missing racial data and four individuals were excluded due to identifying as Native American. An additional 39 individuals were excluded due to missing data on three or more PCEs questions (5 questions in total were asked in parent studies, see below). This resulted in an analytical sample of 2432, with a subset of 560 who completed brain MRI and 281 who completed amyloid PET scans.

The study was conducted according to the guidelines of the Declaration of Helsinki, and approved by the Institutional Review Board of UC Davis (Project IDs: 1121043-13, 960333-36) and the Institutional Review Board of Kaiser (Project IDs: 1278966, 1279068).

### 2.2. Cognitive Assessment Measures

Both KHANDLE and STAR used the Spanish and English Neuropsychological Assessment Scales (SENAS) for cognitive assessment. SENAS is administered in either Spanish or English with primary outcomes categorized into different domains. We included the SENAS verbal episodic memory score, which consisted of a multi-trial word list learning task, as our primary cognitive outcome. We included both baseline and longitudinal measures (i.e., 3 timepoints across a 4-year period) of the SENAS verbal episodic memory score. Details regarding the administration procedures and the development of the SENAS battery are available elsewhere [29,30]. Notably, the SENAS battery was developed to address the need to provide neuropsychological assessment of ethnoculturally and linguistically diverse older adults and scoring is adjusted for age, gender, and education level [31].

Progressive impairment in verbal episodic memory is a hallmark feature of clinical Alzheimer’s Disease but importantly is also associated with anatomical changes within the temporal lobe structures [32]. Given our inclusion of MRI measures of temporal lobe structures (hippocampus), we selected verbal episodic memory as the cognitive outcome of interest.

### 2.3. Positive Childhood Experience Measures

KHANDLE and STAR participants were asked five PCEs questions, with Likert scale 1–5 response options. These include (presented here exactly as worded in studies): (1) How often was there someone in whom you could talk to, trust, and confide? (2) How often was there someone who showed you love and affection? (3) How often was there someone who could help you with your homework? (4) How often was there someone who encouraged and pushed you to succeed in school? (5) How often did you have as much contact as you would like with someone you felt close to, someone in whom you could trust and confide? These variables were highly correlated (Pearson correlation ranged from 0.505 to 0.713) and the internal consistency measures (tested using a Cronbach Alpha) were good (*a* = 0.873, 95% CI = 0.863, 0.882). Thus, the primary analyses were conducted using an average of each individual’s responses across these five questions. Individuals missing data on three or more of these questions were eliminated from the analytic sample (39 individuals, as indicated above). If an individual was missing one or two responses, their missing scores were replaced with an average of their remaining responses. Given the similarities between question one (How often was there someone in whom you could talk to, trust, and confide?) and question five (How often did you have as much contact as you would like with someone you felt close to, someone in whom you could trust and confide?), a sensitivity analysis was conducted following the same procedure outlined above, but with either question five or question one omitted first. Results patterns remained consistent and thus both questions were retained for the final analysis.

### 2.4. MRI Imaging Measures

Randomly selected KHANDLE and STAR participants were invited to participate in brain MRI, which may have occurred at any point during follow-up. MRIs were performed in KHANDLE and STAR using a 3T Siemens (Munich, Germany) Magnetom Trio Syngo system at the University of California, Davis, Imaging Research Center (Sacramento, California). Detailed neuroimaging methods have been described elsewhere [33]. T1-weighted volumetric MP-RAGE (T1: repetition time (TR) = 2500 milliseconds (ms), echo time (TE) = 2.98 ms, inversion time (TI) = 1100 ms, 192 slices total, FOV = 256 mm (mm), acquisition matrix = 256 × 256, slice thickness = 1 mm), fluid attenuated inversion recovery (FLAIR: TR = 8800 ms, TE = 500 ms, TI = 2360 ms, 96 slices total, FOV = 256 mm, acquisition matrix = 256 × 256, slice thickness = 2 mm), and multi-shell diffusion tensor imaging sequences (DTI: TR = 6000 ms, TE = 87 ms, 48 slices total, FOV = 256 mm, acquisition matrix = 96 × 96, slice thickness = 2.7 mm with 2.7 mm gap) were obtained along with diffusion weighted images (13 gradients directions with gradient diffusion sensitivity of b = 500 s/mm^2^, 21 gradients directions with b = 1000 s/mm^2^, 15 gradients directions with b = 2000 s/mm^2^, and 3 images with b = 0 s/mm^2^) followed by brain MRI analysis at the UC Davis Medical Center [33]. Analysis included segmentation and quantification of total cerebral volume via convolutional neuronal network method followed by non-linear co-registration of images to the Desikan atlas for regional gray matter volume calculation [34,35]. Hippocampal volumes were obtained via multi-atlas hippocampal segmentation algorithm to compute hippocampal masks [34].

### 2.5. Amyloid PET Imaging Measures

Amyloid PET scans were also completed for a subset of volunteers in the KHANDLE study (not performed in STAR). Amyloid PET was obtained using ^18^F-Florbetapir (FBP) radiotracer with dynamic acquisition of frames 50–70 min post-injection. FBP-PET was obtained using 10 millicurie (mCi) (370 MBq + 10%) bolus injection of FBP and dynamic acquisition over 20 min (4 × 5 min frames). FBP PET images underwent a quality control assessment and were processed according to methods that have been described elsewhere [36].

Quantification of cortical beta-amyloid was performed using the Freesurfer method (surfer.nmr.mgh.harvard.edu/) [36,37,38] with structural 3T MRI to define cortical regions of interest and reference regions. An “FSR” value was generated for each PET scan through a Freesurfer in-house modification of standard ROIs and this was used as a measure of SUVR. The FSR value was a composite of the anterior and posterior cingulate, frontal, lateral parietal, and lateral temporal regions [37].

### 2.6. Covariates

Covariates in all models included baseline age or imaging age, gender, childhood finances (poor, about average, or well off financially), and adverse childhood experience composite score (ACE). To calculate the ACE score, the method previously used by Gold et al. [1] was implemented. In the KHANDLE and STAR studies, interviewers verbally asked participants whether they had encountered any of nine adverse childhood experiences (ACEs) before the age of 16. These experiences included parental divorce or separation, parental remarriage, witnessing domestic violence, substance abuse by a family member, parental job loss, parental incarceration, serious illness of a family member, the death of their mother, and the death of their father. A total ACE score was calculated by summing the number of experiences reported, with scores ranging from 0 (no ACEs) to 9 (all ACEs reported). For individuals with missing responses on one or more ACE items, missing values were imputed based on the individual’s observed responses. Specifically, if a participant answered six items and endorsed three, the remaining three items were assigned a value of 0.5. Consistent with prior research [1], very few participants reported more than four ACEs, so the total ACE count was capped at 4.

In addition to the above covariates, ethnocultural identity was also controlled for in Models 1 and 2. Indicator dummy variables for White, Latinx, and Asian individuals were entered into the models and individuals racialized as Black served as the reference group. Education was additionally controlled for in all models except for Model 1 in order to establish the relationship between PCEs and episodic memory prior to controlling for the mediator.

### 2.7. Statistical Analysis

**Figure 1:**
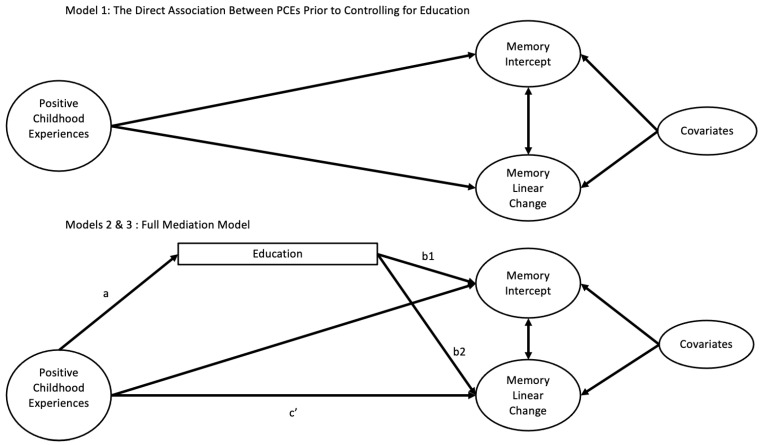
Direct Effects of PCEs and the Mediating Effects of Education Between Intercept and Linear Change in Episodic Memory. “a” represents the effect of the independent variable (PCE) on the mediator (education), “b” is the effect of the mediator (education) on the dependent variable (memory intercept (b1) and slope (b2)), and c’ is the reduced effect of independent variable (PCE) on the dependent variable (memory intercept (b1) and slope (b2)) due to the mediation.

Following the creation of the PCEs variable outlined above, initial descriptive statistics characterized the demographics of (1) the combined sample of KHANDLE and STAR participants; (2) the subset of individuals who received imaging; and (3) individual ethnocultural groups. Subsequent models were tested using structural equation modeling (SEM) with the lavaan package in R [39] within the RStudio IDE (version 1.3.1073; R Core Team, 2023; RStudio Team, 2023; Posit PBC, Boston, MA, USA). All models were estimated using full information maximum likelihood (FIML) under the missing at random assumption. Linear time was coded as 0, 1, and 2. To examine our first aim—the association between PCEs and episodic memory—PCEs served as the independent variable, while episodic memory intercept and linear change (slope) were the dependent variables. Baseline age, gender, childhood financial status, and ACE were included as covariates. To assess whether PCEs had an indirect effect on episodic memory (Aim 2), a conditional indirect effects model was conducted, with PCEs as the independent variable, education as the mediator, and the episodic memory intercept and linear slope as dependent variables. The same covariates—baseline age, gender, childhood financial status, and ACE—were included. The general model structure is summarized in Figure 1. Indirect effects were estimated using bootstrapped standard errors with 1000 iterations and 95% confidence intervals (CIs). This model also allowed for the estimation of direct effects of PCEs and education on memory, while controlling for one another and the covariates. To evaluate whether these relationships varied by ethnocultural group (Aim 3), the mediation model was replicated using a multigroup SEM. However, due to limited within-group change in memory, the model did not converge when estimating linear slope. Consequently, multigroup models were conducted only for the memory intercept. Initially, the model was run with constrained parameters, assuming relationships were equal across groups, followed by an unconstrained model. A chi-square difference test (ANOVA) was significant and other fit indices (CFI, AIC, SRMR) were improved, indicating that the unconstrained model provided a significantly better fit. Thus, results from the unconstrained model are reported. Finally, to examine the association between PCEs and neuroimaging biomarkers of AD/ADRD (hippocampal volume and amyloid burden), linear regression was conducted. Total hippocampal volume and amyloid burden were analyzed as separate dependent variables, with PCEs and education as the primary independent variables. Covariates included age at image acquisition, gender, childhood financial status, and ACE. To assess whether these relationships varied by ethnocultural group, the sample was stratified, and identical regression analyses were conducted within each group.

## 3. Results

### 3.1. Descriptive Statistics

The baseline sample consisted of 2432 participants: 406 Asian, 1179 Black, 349 Latinx, and 498 White individuals (Table 1). A total of 417 participants were lost to follow-up at the second wave (216 Black, 67 White, 68 Latinx, and 66 Asian), with an additional 104 participants lost at the third follow-up (48 Black, 34 White, 15 Latinx, and 7 Asian).

Mean Age at first assessment for the KHANDLE, STAR, MRI, amyloid PET, and combined samples were 76 (SD = 6.7), 69 (SD = 8.8), 72 (SD = 8.0), 75 (SD = 5.8), and 74 (SD = 8.2) years, respectively. Samples ranged from 53–69% female with the combined sample being 62% female. Black individuals were younger (mean age 71, SD = 8.7) with a greater percentage of female (71%) individuals than other ethnocultural groups. The majority of individuals (78%) reported receiving some college education or more. Latinx individuals reported the lowest levels of education (mean education 4.7, SD = 1.70). Asian individuals reported the lowest levels of PCEs on average (mean PCEs = 3.5, SD = 1.0). Missing data for baseline covariates was minimal for the current study with no missing data across age and gender. Further, our quantification of ACE led to imputation if there were missing data. Additionally, missing data in the quantification of ACE were addressed through imputation. However, 146 participants (53 Black, 43 White, 30 Latinx, and 20 Asian) did not report childhood finances.

### 3.2. Direct Effects of PCEs and Education on Memory

*Intercepts*. Prior to examining the indirect associations of education through PCEs, we first sought to assess the direct associations of PCEs on memory within the whole sample and each racial group (Table 2). Model 1 examined the direct association within the whole sample prior to adding in education. Model 2 added education. The findings showed that PCEs were significantly and positively associated with memory intercept (b = 0.033; 95% CI: 0.003, 0.064, Beta = 0.048; Model 1). In Model 2, PCEs were no longer associated with memory intercept. Education was significantly and positively associated with memory intercept (b = 0.185; 95% CI: 0.151, 0.218, Beta = 0.234; Model 2). Model 1 explained 33% of the variance in memory intercept and Model 2 explained 36%.

*Linear Slope*. Findings for the direct association between PCEs and education on memory intercept can be found in Table 3. PCEs were positively associated with the linear slope in Model 1 (b = 0.019; 95% CI: 0.000, 0.036, Beta = 0.116; Model 1) and Model 2 (b = 0.020; 95% CI: 0.001, 0.038, Beta = 0.121; Model 2). Education was not associated with change in memory. Model 1 explained 6.6% of the variance in the memory intercept and Model 2 explained 6.7%.

*Multigroup Intercept Model.* The multigroup models enabled us to examine the relationships between PCEs, education, and memory outcomes across ethnocultural groups (Table 4). Given the embedded structure of this model, unstandardized coefficients can be directly compared across groups. While standardized coefficients (beta weights) are also reported to provide a sense of effect magnitude, they should not be compared between groups, as differences in sample size and variance can influence standardized values. Our results showed that PCEs were not associated with memory intercept across any racial group after controlling for education. Education was significantly and positively associated with memory in Black (b = 0.160; 95% CI: 0.117, 0.209, Beta = 0.200; Model 3), White (b = 0.246; 95% CI: 0.180, 0.313, Beta = 0.298; Model 3), Latinx (b = 0.124; 95% CI: 0.051, 0.194, Beta = 0.184; Model 3), and Asian (b = 0.195; 95% CI: 0.100, 0.290, Beta = 0.201; Model 3) participants. Among Black, White, Latinx, and Asian participants respectively, 39%, 45%, 37%, and 34% of the variance in the memory intercept was explained. Memory linear slope could not be examined due to lack of change within individual groups.

### 3.3. Indirect Effects of Education Through PCEs

Prior to examining the mediation, the a path (PCEs association with education) was examined (Table 5). PCEs were significant and positively associated with education across racial groups and within the combined sample, with the smallest effect being within Black participants. There was a significant positive indirect effect of education through PCEs within the whole sample for the memory intercept (b = 0.023; 95% CI: 0.015, 0.032, Beta = 0.034; Model 2). The indirect effect for linear slope was not significant within the whole sample. These findings were consistent in Black (b = 0.012; 95% CI: 0.004, 0.023, Beta = 0.017; Model 3), White (b = 0.041; 95% CI: 0.019, 0.066, Beta = 0.055; Model 3), Latinx (b = 0.030; 95% CI: 0.011, 0.051, Beta = 0.050; Model 3), and Asian (b = 0.035; 95% CI: 0.015, 0.032, Beta = 0.034; Model 3) participants. The total association of education (direct plus indirect) was significant within the whole sample for the memory intercept (b = 0.035; 95% CI: 0.004, 0.064, Beta = 0.050; Model 2) and slope (b = 0.019; 95% CI: 0.001, 0.038, Beta = 0.118; Model 2). The total association of education was also significant and positive for Latinx (b = 0.075; 95% CI: 0.010, 0.135, Beta = 0.124; Model 3) and Asian (b = 0.089; 95% CI: 0.017, 0.163, Beta = 0.119; Model 3), but not for Black or White participants.

### 3.4. The Association Between PCEs and Education on Hippocampal Volume and Amyloid Burden

Results examining the association between PCEs, hippocampal volume, and amyloid burden are shown in Table 6 and Table 7. PCEs were not associated with hippocampal volume or amyloid burden in the combined sample or within any of the individual racial groups. Education was positively associated with hippocampal volume in the combined sample (b = 0.102; 95% CI: 0.006, 0.197, Beta = 0.084; Model 4) and within Asian participants (b = 0.276; 95% CI: 0.042, 0.509, Beta = 0.241; Model 4). Education was not associated with amyloid volume in the combined sample or within any racial group.

## 4. Discussion

In our study of ethnoculturally diverse older adults, we found that, in the combined sample, more self-reported PCEs were associated with higher level and more change in verbal episodic memory prior to controlling for education. After entering education into the model, PCEs were no longer associated with memory intercept, but their relationship with memory change persisted. Education was positively associated with memory intercept but was not associated with change. Our multigroup analyses demonstrated similar pattern of results between groups. Education was positively associated with memory intercept in all groups and PCEs were not associated with memory after controlling for education. PCEs significantly and positively predicted education in the combined sample and within each ethnocultural group. Notably, the relationship between PCEs and education was weaker among Black participants. There was a significant indirect effect of PCEs through education for memory intercept for the combined sample and for all ethnocultural groups. There was no indirect effect for linear slope. We found no association in the combined sample or in the stratified samples between PCEs and hippocampal volume. Education was positively associated with hippocampal volume in the combined sample and within Asian participants. Neither PCEs nor education predicted amyloid burden in the combined samples or in any of the ethnocultural groups. Our study builds on the limited research examining PCEs and late-life cognition and is the first to investigate education as a mediator while assessing the association longitudinally. This study is additionally unique in our examination of imaging biomarkers of AD/ADRD among ethnoculturally diverse individuals. Our findings suggest that PCEs promote better cognition as individuals enter older adulthood through educational attainment, though relationships between PCEs and cognition may be complex.

Lee et al. [12] found that those who reported exposure to happy family homes during childhood had better late-life cognitive functioning (as measured by a MOCA translated outcome score) than those who had less exposure to happy family homes during childhood. The operationalization of PCEs in their study was quite different from ours; however, both findings may suggest that happier and more supportive home environments may promote later achievement and resource accumulation in adulthood (e.g., greater educational attainment, social connectedness, self-mastery, and health). Prior work in neuroscience has also shown exposure to positive emotions, as can be created by PCEs, has potential to increase prefrontal cortex activity, with early life brain activity and structure carrying over into adulthood, impacting one’s ultimate cognitive reserve development [40,41]. Exposure to PCEs can promote social connectedness, which builds reserve into adulthood through relationships that place cognitive demands on individuals and promote mental stimulation [42]. Increased sense of self-mastery and control, which has been shown to have a positive impact on cognition through better coping of stress, is also associated with greater reserve [43].

Our findings suggest that PCEs may be associated with cognitive change; however, the effect sizes were very small, and the models explained only 7% of the variance in cognitive change. Given our mediation results, which indicate that PCEs may exert their protective effects through education, along with prior research demonstrating an association between PCEs and educational success [23], our findings align with past studies suggesting that education has a stronger influence on cognitive level rather than cognitive change [44]. Our analyses may have been limited by the characteristics of the cohort, as most individuals in KHANDLE and STAR did not have dementia at baseline, resulting in minimal cognitive change and memory change over the 4-year period. Our study is among the few to examine the association between PCEs and cognitive change, highlighting the need for further research over longer time frames to determine whether PCEs interact with other cognitive reserve factors or have a distinct impact on cognitive decline. Cognitive reserve is one mechanism by which individuals are protected from significant cognitive decline leading to dementia, even in the presence of neurodegenerative pathology. Reserve is the result of greater resilience of neuronal networks built through multiple activities and sociobehavioral experiences (like positive environments) that protect cognition [45,46].

Our multigroup models demonstrated relatively similar patterns, in that there was a positive indirect effect of PCEs through education but PCEs were not associated with memory above and beyond education. Effect sizes in these analyses were relatively similar, but the strongest effects were in Asian and Latinx participants. These findings are promising, as they suggest that programs promoting PCEs may help reduce cognitive disparities observed in older adulthood. However, when examining the a path or the association between PCEs and education, the association was weakest among Black participants. A previous study demonstrated that approximately 38% of individuals in the STAR sample attended a segregated school at some point between 1st and 12th grades [47], which is an important consideration for educational quality across groups. It is therefore likely that other factors including racial biases and discrimination, may be reducing the strength of this relationship.

Previous work has demonstrated adverse childhood events and lack of social support may be associated with hippocampal volume in adults [48,49]; however, there is absence of work examining association between PCEs and more detailed imaging biomarkers in older individuals. We believe the current study is the first to examine the association between PCEs and amyloid PET burden. We did not find an association between ACEs or PCEs and hippocampal volume or amyloid burden. Education was positively associated with hippocampal volume in the combined sample, however, when stratified this relationship was only significant in Asian participants. It is difficult to say why this association persisted in Asian individuals and more research is needed to determine why education did not have the neuroanatomical benefits for other ethnocultural groups in our sample.

Limitations of our study include generalizability of results. The current study was conducted in a positively selected sample that had access to health care through the Kaiser Permanente system. In the United States, many individuals do not have access to health care, so our findings cannot be generalized to those individuals. These individuals are often more socioeconomically disadvantaged than others, thus applying findings from our work to the most highly disadvantaged groups may not be possible. Prior work, however, has demonstrated that members of the Kaiser Permanente care system are similar across demographic and socioeconomic characteristics to the general population within each of the regions where Kaiser Permanente offers care, suggesting our findings are generalizable to some degree even given the above caveat [50].

We operationalized PCEs using 5-point Likert scale questions and lacked additional measures of childhood support and social experiences. The PCE rating scale data was drawn from parent studies and was limited to 5 questions. We did not have the ability to change the phrasing of any of the questions or expand them. Ideally, we would have had access to a more robust and standardized measure of PCEs. However, Bethell et al. [15] previously examined the associations between PCEs and adult mental and relational health in a statewide sample of individuals with varying sociodemographic and ethnocultural characteristics using a 7-item measure of PCEs that overlap with some of the 5 questions used in KHANDLE and STAR. Bethell et al. adapted their 7-item measure of PCEs from 4 subscales included in the Child and Youth Resilience Measure–28, which was designed for cultural sensitivity [51]. As with all retrospective studies, the integrity of memories of childhood and recall bias might also be questioned; however, previous work has shown that early life autobiographical memories are relatively highly preserved even among older adults in population-based studies [52]. Available imaging biomarker data was also limited to what was available from parent studies, thus there was no ability to perform more advanced imaging biomarker analyses. Finally, the current study consisted of a relatively short 4-year longitudinal follow-up in a healthy cohort. It may be that more time is needed to allow for more variance in cognitive change to truly assess the relationship with PCEs. Strengths of our study include the ethnocultural diversity of participants, longitudinal data, and combination of high-quality cognitive assessments with neuroimaging data.

It is important to emphasize that race and ethnicity are not biological variables and should not be viewed as such in this project. Though there is debate regarding a biological base to race and ethnicity, it is important to consider that racial and ethnic categories used in research in the United States were developed by the Office of Management and Budget (OMB) based on an arbitrary framework never designed to be interpreted as scientific or anthropologic in nature [53]. Ethnocultural identity is complex and encapsulates numerous variables that represent lived experiences, exposure to systemic injustices, and other life course experiences. Inequities between racial and ethnic groups clearly exist; however, continued measurement of these variables as social categories, not biological variables, remains important.

## 5. Conclusions

The current study found that positive childhood experiences lead to better verbal episodic memory in late-life through their association with educational attainment. This pattern is relatively stable between ethnocultural groups, though systemic barriers likely weakened this association (PCEs with education) in Black participants. Our study is one of the first to investigate the impact of PCEs on both cognitive and imaging outcomes in older adulthood among ethnoculturally diverse individuals. This largely unexplored area of research needs further investigation, given risk and resilience factors occur on a continuum throughout the lifespan and extend beyond measures like socioeconomic status and education, which have been heavily emphasized. Early childhood support interventions through policies that ameliorate adverse childhood experiences, while also bolstering PCEs, could potentially impact late-life cognition, combat systemic disadvantages, and promote disease prevention. Some efforts already undertaken to bolster childhood experiences include pediatric primary care settings offering opportunities for improved relational health and behavioral modeling strategies for parents. Greater resources dedicated to pediatric primary care settings could help further this impact [54,55]. Additional initiatives that are place-based (focused within a community) and integrate health care providers, early childhood educators, local community resources, and community members can also promote PCEs, particularly within historically marginalized communities [55].

## Figures and Tables

**Table 1:** Descriptive Statistics for Participants Included from the KHANDLE and STAR Samples and Individuals who Received MRI and PET Imaging.

	KHANDLE	STAR	MRI Sample		PET Sample		Total
Mean Age at First Assessment (SD)	76 (6.7)	69 (8.8)	72 (8.0)		75 (5.8)		74 (8.2)
% Female	59	69	60		53		62
Race, N (%)							
Black	436 (26)	743 (99)	298 (53)		50 (18)		1179 (48)
White	498 (30)	0	93 (17)		84 (30)		498 (20)
Latinx	343 (20)	6 (1)	93 (17)		80 (28)		349 (14)
Asian	406 (24)	0	76 (14)		67 (24)		406 (17)
Education, N (%)							
Grade School	50 (3)	2 (0)	3 (1)		3 (1)		52 (2)
Some High School	63 (4)	17 (2)	14 (3)		8 (3)		80 (3)
Tech/Trade School	75 (4)	31 (4)	17 (3)		8 (3)		106 (4)
High School Graduate	167 (10)	114 (15)	59 (11)		31 (13)		281 (12)
Some College	576 (33)	332 (44)	207 (27)		88 (38)		838 (34)
College Graduate	424 (24)	130 (17)	141 (25)		32 (14)		554 (23)
Graduate School	387 (22)	133 (18)	119 (22)		61 (26)		520 (21)
Total N	1683	749	560		283		2432
	Ethnocultural Group Means						
	Black	White		Latinx		Asian	
Mean Age (SD)	71 (8.7)	77 (7.2)		76 (6.5)		76 (6.6)	
% Female	68	58		59		53	
Education (SD)	5.2 (1.3)	5.5 (1.4)		4.7 (1.7)		5.8 (1.2)	
Childhood Finances	0.726 (0.604)	0.892 (0.555)		0.665 (0.627)		0.762 (0.633)	
ACE	1.81 (1.40)	1.45 (1.36)		1.85(1.41)		0.980 (1.14)	
PCEs (SD)	4.1 (0.9)	4.1 (0.9)		3.4 (1.2)		3.5 (1.0)	
Education (SD)	5.2 (1.3)	5.5 (1.4)		4.7 (1.7)		5.8 (1.2)	
Verbal Episodic Memory (SD)	0.02 (0.9)	0.06 (0.9)		−0.17 (0.9)		0.09 (0.9)	

SD—Standard deviation, MRI—Magnetic Resonance Imaging, PET—Positron emission tomography. Education was coded as: 1 = grade school, 2 = some high school, 3 = tech or trade school, 4 = high school graduate or GED, 5 = some college, 6 = college, 7 = graduate school.

**Table 2:** Intercept of Episodic Memory Regressed on PCEs, Education (Model 2), Age, Gender, Childhood Finances, ACE, and Ethnocultural Group.

	Model 1				Model 2			
	b Weight (95% CI)	Std. Beta	SE	*p* Value	b Weight (95% CI)	Std. Beta	SE	*p* Value
Age	−0.357 (−0.390, −0.324)	−0.485	0.017	0.000	−0.334 (−0.368, −0.304)	−0.459	0.017	0.000
Gender	0.465 (0.406, 0.527)	0.309	0.032	0.000	0.487 (0.421, 0.545)	0.327	0.032	0.000
Childhood Finances	−0.001 (−0.044, 0.045)	−0.001	0.022	0.970	−0.013 (−0.055, 0.029)	−0.014	0.022	0.549
ACE	0.003 (−0.020, 0.027)	0.006	0.012	0.797	0.005 (−0.018, 0.028)	0.009	0.012	0.673
White	0.336 (0.251, 0.431)	0.186	0.045	0.000	0.269 (0.186, 0.354)	0.151	0.042	0.000
Latinx	0.096 (−0.003, 0.188)	0.046	0.047	0.040	0.117 (0.026, 0.209)	0.057	0.046	0.010
Asian	0.393 (0.298, 0.499)	0.201	0.051	0.000	0.296 (0.195, 0.402)	0.153	0.050	0.000
PCEs	0.033 (0.003, 0.064)	0.048	0.016	0.035	0.011 (−0.019, 0.040)	0.016	0.015	0.460
Education	--	--	--	--	0.185 (0.151, 0.218)	0.234	0.017	0.000
R Squared	0.330				0.364			

SE = Standard Error.

**Table 3:** Linear Slope of Episodic Memory Regressed on PCEs, Education (Model 2), Age, Gender, Childhood Finances, ACE, and Ethnocultural Group.

	Model 1				Model 2			
	b Weight (95% CI)	Std. Beta	SE	*p* Value	b Weight (95% CI)	Std. Beta	SE	*p* Value
Age	−0.014 (−0.034, 0.007)	−0.083	0.010	0.149	−0.016 (−0.036, 0.004)	−0.096	0.010	0.108
Gender	0.024 (−0.012, 0.059)	0.068	0.018	0.202	0.024 (−0.011, 0.063)	0.069	0.018	0.188
Childhood Finances	−0.021 (−0.044, 0.002)	−0.099	0.012	0.085	−0.021 (−0.043, 0.002)	−0.098	0.012	0.074
ACE	−0.005 (−0.018, 0.008)	−0.041	0.007	0.464	−0.005 (−0.017, 0.008)	−0.041	0.006	0.445
White	0.053 (0.004, 0.102)	0.127	0.024	0.028	0.057 (0.011, 0.106)	0.137	0.024	0.019
Latinx	0.092 (0.037, 0.144)	0.190	0.028	0.001	0.091 (0.038, 0.143)	0.190	0.028	0.001
Asian	−0.021 (−0.072, 0.032)	−0.046	0.027	0.445	−0.016 (−0.070, 0.038)	−0.036	0.027	0.554
PCEs	0.019 (0.000, 0.036)	0.116	0.009	0.039	0.020 (0.001, 0.038)	0.121	0.009	0.037
Education	--	--	--	--	−0.004 (−0.026, 0.016)	−0.021	0.011	0.730
R Squared	0.066				0.067			

SE = Standard Error.

**Table 4:** Stratified Analyses of Intercept of Episodic Memory Regressed on PCEs, Education (Model 2), Age, Gender, Childhood Finances, ACE.

		b Weight (95% CI)	Std. Beta	SE	*p* Value
Black	Age	−0.313 (−0.353, −0.278)	−0.489	0.018	0.000
	Gender	0.461 (0.388, 0.540)	0.315	0.039	0.000
	Childhood Finances	−0.020 (−0.071, 0.030)	−0.024	0.026	0.428
	ACE	0.002 (−0.025, 0.030)	0.003	0.014	0.911
	PCEs	0.019 (−0.020, 0.055)	0.026	0.019	0.322
	Education	0.160 (0.117, 0.209)	0.200	0.023	0.000
	R Squared	0.390			
White	Age	−0.418 (−0.482, −0.351)	−0.464	0.034	0.000
	Gender	0.567 (0.445, 0.688)	0.365	0.061	0.000
	Childhood Finances	−0.022 (−0.124, 0.070)	−0.021	0.048	0.641
	ACE	0.018 (−0.027, 0.067)	0.032	0.024	0.446
	PCEs	0.021 (−0.038, 0.083)	0.028	0.031	0.496
	Education	0.246 (0.180, 0.313)	0.298	0.033	0.000
	R Squared	0.451			
Latinx	Age	−0.401 (−0.500, −0.305)	−0.438	0.051	0.000
	Gender	0.478 (0.337, 0.621)	0.339	0.072	0.000
	Childhood Finances	−0.135 (−0.223, −0.039)	−0.159	0.048	0.005
	ACE	−0.024 (−0.073, 0.031)	−0.049	0.027	0.366
	PCEs	0.045 (−0.018, 0.104)	0.074	0.032	0.158
	Education	0.124 (0.051, 0.194)	0.184	0.037	0.001
	R Squared	0.371			
Asian	Age	−0.375 (−0.473, −0.278)	−0.372	0.050	0.000
	Gender	0.586 (0.449, 0.741)	0.374	0.074	0.000
	Childhood Finances	0.035 (−0.054, 0.124)	0.037	0.046	0.446
	ACE	−0.002 (−0.070, 0.062)	−0.003	0.034	0.949
	PCEs	0.053 (−0.020, 0.127)	0.071	0.038	0.155
	Education	0.195 (0.100, 0.290)	0.201	0.048	0.000
	R Squared	0.342			

SE = Standard Error.

**Table 5:** Education Regressed on PCEs (Path A); Education as Mediator Between PCEs and Episodic Memory.

Association Between PCE and Education (a Path)				
	b Weight (95% CI)	Std. Beta	SE	*p* Value
Asian	0.182 (0.099, 0.260)	0.236	0.042	0.000
White	0.168 (0.079, 0.256)	0.184	0.045	0.000
Latinx	0.245 (0.150, 0.331)	0.272	0.046	0.000
Black	0.077 (0.023, 0.130)	0.087	0.027	0.005
Combined Sample	0.127 (0.089, 0.163)	0.146	0.019	0.000
Total Indirect Effect of Education Through PCEs				
Black	0.012 (0.004, 0.023)	0.017	0.005	0.011
White	0.041 (0.019, 0.066)	0.055	0.012	0.001
Latinx	0.030 (0.011, 0.051)	0.050	0.010	0.003
Asian	0.035 (0.015, 0.059)	0.047	0.012	0.002
Combined Sample Intercept	0.023 (0.015, 0.032)	0.034	0.004	0.000
Combined Sample Linear Slope	0.000 (−0.003, 0.002)	−0.003	0.001	0.734
Total Effect of Education				
Black	0.031 (−0.008, 0.069)	0.044	0.019	0.111
White	0.062 (−0.001, 0.126)	0.083	0.033	0.062
Latinx	0.075 (0.010, 0.135)	0.124	0.032	0.019
Asian	0.089 (0.017, 0.163)	0.119	0.037	0.017
Combined Sample Intercept	0.035 (0.004, 0.064)	0.050	0.016	0.027
Combined Sample Linear Slope	0.019 (0.001, 0.038)	0.118	0.009	0.041

SE = Standard Error.

**Table 6:** Linear Regressions Predicting Hippocampal Volume in the Full Sample and Stratified by Race, Adjusting for Age at Imaging and Gender.

		b Weight (95% CI)	Std. Beta	SE	*p* Value
Black	Age at MRI	−0.032 (−0.044, −0.020)	−0.293	0.006	0.000
	Gender	−0.562 (−0.777, −0.348)	−0.286	0.109	0.000
	Childhood Finances	−0.098 (−0.233, 0.036)	−0.084	0.068	0.152
	ACE	−0.048 (−0.123, 0.027)	−0.072	0.038	0.207
	PCEs	−0.097 (−0.212, 0.018)	−0.096	0.058	0.099
	Education	0.105 (−0.024, 0.233)	0.089	0.065	0.111
	R Squared	0.1535			
White	Age at MRI	−0.049 (−0.084, −0.014)	−0.267	0.018	0.007
	Gender	−0.998 (−1.426, −0.570)	−0.441	0.215	0.000
	Childhood Finances	−0.025 (−0.365, 0.314)	−0.015	0.171	0.882
	ACE	0.102 (−0.069, 0.273)	0.117	0.086	0.239
	PCEs	0.142 (−0.070, 0.354)	0.125	0.107	0.188
	Education	0.013 (−0.248, 0.274)	0.010	0.131	0.921
	R Squared	0.2472			
Latinx	Age at MRI	−0.062 (−0.100, −0.024)	−0.348	0.019	0.002
	Gender	−0.517 (−0.943, −0.092)	−0.252	0.214	0.018
	Childhood Finances	−0.101 (−0.377, 0.176)	−0.081	0.139	0.470
	ACE	0.013 (−0.135, 0.161)	0.019	0.074	0.861
	PCEs	0.167 (−0.041, 0.375)	0.182	0.104	0.114
	Education	0.053 (−0.215, 0.322)	0.045	0.135	0.693
	R Squared	0.1671			
Asian	Age at MRI	−0.061 (−0.091, −0.030)	−0.385	0.015	0.000
	Gender	−0.668 (−1.012, −0.324)	−0.381	0.172	0.000
	Childhood Finances	−0.022 (−0.223, 0.180)	−0.021	0.101	0.831
	ACE	0.062 (−0.068, 0.193)	0.095	0.065	0.344
	PCEs	0.033 (−0.134, 0.200)	0.041	0.083	0.692
	Education	0.276 (0.042, 0.509)	0.241	0.117	0.021
	R Squared	0.3799			
Combined Sample	Age at MRI	−0.038 (−0.048, −0.027)	−0.311	0.005	0.000
	Gender	−0.656 (−0.812, −0.500)	−0.324	0.079	0.000
	Childhood Finances	−0.083 (−0.184, 0.019)	−0.066	0.052	0.109
	ACE	0.000 (−0.057, 0.056)	0.000	0.029	0.991
	White	0.617 (0.385, 0.849)	0.231	0.118	0.000
	Latinx	0.550 (0.316, 0.784)	0.202	0.119	0.000
	Asian	0.509 (0.258, 0.760)	0.176	0.128	0.000
	PCEs	0.022 (−0.058, 0.102)	0.023	0.041	0.586
	Education	0.102 (0.006, 0.197)	0.084	0.049	0.037
	R Squared	0.2231			

SE = Standard Error.

**Table 7:** Linear Regressions Predicting Amyloid Burden in the Full Sample and Stratified by Race, Adjusting for Age at Imaging and Gender.

		b Weight (95% CI)	Std. Beta	SE	*p* Value
Black	Age at PET	−0.020 (−0.063, 0.023)	−0.156	0.021	0.349
	Gender	0.285 (−0.196, 0.766)	0.192	0.238	0.237
	Childhood Finances	−0.097 (−0.406, 0.211)	−0.108	0.152	0.528
	ACE	0.010 (−0.141, 0.162)	0.022	0.075	0.889
	PCEs	−0.136 (−0.384, 0.112)	−0.176	0.123	0.274
	Education	−0.066 (−0.347, 0.214)	−0.079	0.138	0.634
	R Squared	0.034			
White	Age at PET	−0.002 (−0.051, 0.048)	−0.008	0.025	0.949
	Gender	−0.087 (−0.671, 0.498)	−0.034	0.293	0.769
	Childhood Finances	−0.131 (−0.587, 0.325)	−0.066	0.229	0.569
	ACE	−0.271 (−0.505, −0.038)	−0.268	0.117	0.023
	PCEs	−0.006 (−0.300, 0.289)	−0.004	0.148	0.970
	Education	−0.342 (−0.713, 0.029)	−0.218	0.186	0.070
	R Squared	0.025			
Latinx	Age at PET	0.006 (−0.033, 0.045)	0.042	0.019	0.750
	Gender	−0.081 (−0.518, 0.355)	−0.047	0.219	0.711
	Childhood Finances	0.153 (−0.124, 0.429)	0.145	0.139	0.275
	ACE	−0.050 (−0.205, 0.105)	−0.084	0.078	0.523
	PCEs	−0.014 (−0.216, 0.189)	−0.018	0.101	0.894
	Education	−0.170 (−0.450, 0.110)	−0.171	0.140	0.231
	R Squared	0.037			
Asian	Age at PET	−0.004 (−0.043, 0.034)	−0.030	0.019	0.819
	Gender	0.206 (−0.220, 0.633)	0.129	0.213	0.337
	Childhood Finances	−0.054 (−0.297, 0.190)	−0.058	0.121	0.660
	ACE	−0.100 (−0.268, 0.067)	−0.160	0.084	0.236
	PCEs	−0.082 (−0.284, 0.120)	−0.112	0.101	0.420
	Education	0.244 (−0.044, 0.532)	0.232	0.144	0.095
	R Squared	0.028			
Combined Sample	Age at PET	0.001 (−0.020, 0.023)	0.008	0.011	0.900
	Gender	0.043 (−0.202, 0.288)	0.021	0.124	0.730
	Childhood Finances	−0.012 (−0.173, 0.149)	−0.010	0.082	0.883
	ACE	−0.087 (−0.177, 0.004)	−0.122	0.046	0.061
	White	0.341 (−0.036, 0.719)	0.157	0.192	0.076
	Latinx	−0.063 (−0.443, 0.316)	−0.028	0.193	0.743
	Asian	−0.220 (−0.618, 0.177)	−0.095	0.202	0.277
	PCEs	−0.015 (−0.134, 0.103)	−0.017	0.060	0.801
	Education	−0.106 (−0.260, 0.047)	−0.090	0.078	0.172
	R Squared	0.029			

## Data Availability

The data that support the findings of this study are available from the Kaiser Study of Healthy Aging and Diverse Life Experiences (KHANDLE) and Study of Healthy Aging in African Americans (STAR) studies. Restrictions apply to the availability of these data. Data were obtained from the KHANDLE and STAR studies and are available from these studies directly with the permission of the study teams.
